# Supplementary material for: Detrimental network effects in privacy: A graph-theoretic model for node-based intrusions
Source: Patterns (N Y). 2023 Jan 13;4(1):100662. doi: 10.1016/j.patter.2022.100662 (PMC9868678; doi:10.1016/j.patter.2022.100662)
Supplement: Document S1. Supplemental experimental procedures and Figures S1 and S2 [file mmc1.pdf]

Patterns, Volume *4*

Supplemental information

Detrimental network effects in privacy: A graph-theoretic model for node-based intrusions

Florimond Houssiau, Piotr Sapieżyński, Laura Radaelli, Erez Shmueli, and Yves-Alexandre de Montjoye

Supplemental Experimental Procedures

***S1**: Alternatives to uniform node intrusions*

In this paper, we study edge- and node-observability through uniform node-based intrusions. While this assumption is realistic for the three use cases we present, other non-uniform attacks would be interesting to investigate. Most of our mathematical definitions do not rely on this uniformity assumption and the framework can be easily extended with different compromised nodes distribution or targeting. For instance, targeted attacks to observe the entire network using as few nodes as possible have been studied before [1, 2]. These differ from our work in that the attacker knows the structure of entire network, and chooses nodes to compromise in order to learn all of the nodes' private states (e.g. in a power grid).

***S2**: Facebook's node-observability*

Our analysis of Facebook's node-observability is based on the degree distribution of Facebook users in the United States in 2011 [3]. We shift and truncate this distribution so that the average degree matches the 2014's average degree (which has been published [4]). Formally, let $P_{2011}(d)$ be the distribution of degrees in 2011, such that $\sum_{d=0}^{5000} P_{2011}(d) = 1$. This distribution is truncated at 5000 because Facebook doesn't allow a user to have more than that number of friends. We compute an estimate degree distribution for 2014 as:

$$\widehat{P}_{2014}(d) = \begin{cases} 0 & \text{if } d < \sigma \\ A \cdot P_{2011}(d - \sigma) & \text{otherwise} \end{cases}$$

here the shift σ is chosen such that the average degree $\sum_{d=0}^{5000} d\widehat{P}_{2014}(d)$ matches the empirical value, and the scaling constant A is such that $\widehat{P}_{2014}$ sums to 1. This is a simple approximation, assuming that users uniformly increased their friends count between 2011 and 2014.

We convert the distribution $\widehat{P}_{2014}$ to absolute degree counts by multiplying by $N = 205 \cdot 10^6$, the number of Facebook users in the USA [5]. We then use theorem 3 to compute the 1-hop node-observability from the degree counts. Our analysis shows that compromising $n_C = 270,000$ profiles, as Cambridge Analytica did, allows an attacker to observe 68.0 million users, giving a node-observability of 0.318.

To study the 2-hops observability of the Facebook graph, we generate synthetic graphs from the distribution $\hat{P}_{2014}$ using the configuration model [6]. Generating such a graph with $N = 205 \cdot 10^6$ nodes is impractical, we compute the observability curve for graphs with $N' < N$ nodes, with N' ranging from 10^5 to $2 \cdot 10^6$, shown in Fig. 1. Our results show that compromising a fraction $\frac{n_{CA}}{\text{US population}} = \frac{270000}{205 \cdot 10^6} \approx 0.0013$ of the network would leads to a node-observability of 1.0 on all the synthetic graphs. This analysis strongly suggests that a 2-hop policy would have enabled Cambridge Analytica to observe the whole Facebook network.

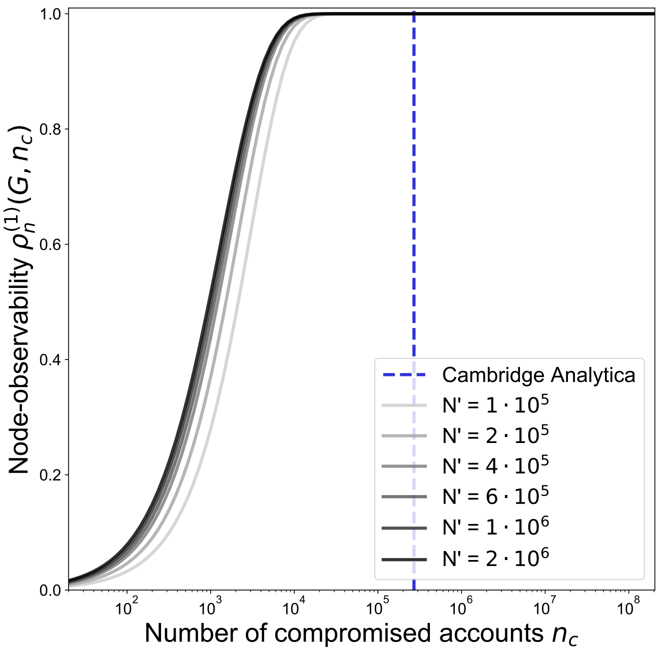

Figure 1: **Estimation of Cambridge Analytica's attack success if Facebook allowed for 2-hop**. 2-hop node-observability of graphs generated using the configuration model with degree distribution similar to that of the shifted Facebook degree distribution $\hat{P}_{2014}$ (solid curve). The curves correspond to graphs with different number of nodes N' smaller than $N = 205 \cdot 10^6$, the number of Facebook users in the USA in 2014. The curves quickly converge for growing n', and all agree for $n_p = 270,000$ (Cambridge Analytica).

S3: Close proximity Networks

The first step of our computation of the observability of cities is to estimate the average probability of observation from a fixed number of sensors in a 1 km^2 cell. We use Algorithm 1 to estimate this from geo-tagged hourly co-location data, which we model as a function of the hourly time-stamp (in a set of hours $\mathcal{T}$) and the cell (from a set of 1 km^2 cells $\mathcal{C}$) to the set of all graphs (denoted by $\mathbb{G}$) $\mathbb{G} : \mathcal{T} \times \mathcal{C} \rightarrow \mathbb{G}$.

Algorithm 1 Average Probability of Observation from hourly co-location data

procedure AVGPROBAOBS($n_p, \mathbb{G}, n_{samples}$)
    $observed \leftarrow 0$
    for $i = 1, \ldots, n_{samples}$ **do**
        Pick a cell uniformly at random $c \leftarrow \mathcal{C}$;
        Pick a time uniformly at random $h \leftarrow \mathcal{T}$;
        Define $G(c, h) = (V, E)$, pick n_p nodes uniformly at random from V, V_p;
        Pick a target u uniformly at random from non-infected nodes $u \leftarrow V \backslash V_p$;
        if there is an edge between u and a node in V_p **then**
            $observed += 1$
        end if
        $m_i \leftarrow x \cdot B_i$
    end for
    return $observed/n_{samples}$
end procedure

We use procedure AvgProbaObs with n_p ranging from 5 to 385, in increments of 5, with $n_{samples} = 25$ (about 10000 total samples). Fig. 2 presents our results, along with the fit by $\hat{\mu}(n_p) = 0.13 \log(n_p) - 0.05$ ($R^2 = 0.876$).

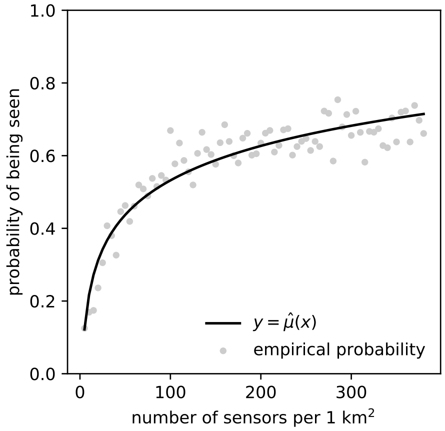

Figure 2: **Average probability of observation in hourly co-location graphs in a** $1\ km^2$ **cell.** Each dot is obtained from AvgProbaObs on the DTU dataset, using $n_{samples} = 25$. The solid curve is $\hat{\mu}(n_p) = 0.13 \log(n_p) - 0.05$, which fits the observed data ($R^2 = 0.876$).

References

[1] Yang Yang, Jianhui Wang, and Adilson E Motter. Network observability transitions. *Physical Review Letters*, 109(25):258701, 2012.

[2] Takehisa Hasegawa, Taro Takaguchi, and Naoki Masuda. Observability transitions in correlated networks. *Physical Review E*, 88(4):042809, 2013.

[3] Johan Ugander, Brian Karrer, Lars Backstrom, and Cameron Marlow. The anatomy of the facebook social graph. *arXiv preprint arXiv:1111.4503*, (2011).

[4] Aaron Smith. What people like and dislike about facebook, February 2014.

[5] Statista. Number of monthly active facebook users in the united states and canada as of 4th quarter 2018 (in millions)., 2018.

[6] Mark EJ Newman. The structure and function of complex networks. *SIAM review*, 45(2):167–256, 2003.
