# Supplementary material for: Detrimental network effects in privacy: A graph-theoretic model for node-based intrusions
Source: Patterns (N Y). 2023 Jan 13;4(1):100662. doi: 10.1016/j.patter.2022.100662 (PMC9868678; doi:10.1016/j.patter.2022.100662)
Supplement: Document S2. Article plus supplemental information [file mmc2.pdf]

Article

Patterns

Detrimental network effects in privacy: A graph-theoretic model for node-based intrusions

Highlights

- We propose a framework to model the reach of networked data collection
- The framework relies on the notions of node- and edge-observability
- We independently confirm the number of accounts observed by Cambridge Analytica
- We evaluate the reach of surveillance in mobile phone and close-proximity networks

Authors

Florimond Houssiau,
Piotr Sapieżyński, Laura Radaelli,
Erez Shmueli,
Yves-Alexandre de Montjoye

Correspondence

demontjoye@imperial.ac.uk

In brief

Proportionality is one of the tenets of data protection laws. Yet, we currently lack a robust analytical framework to evaluate the reach of modern data collections. Here, we propose a graph-theoretic model and notions of node- and edge-observability to quantify the reach of networked data collections. We prove closed-form expressions for our metrics, independently validate the number of profiles collected by Cambridge Analytica, and evaluate the reach of surveillance in mobile phone networks and close-proximity tracking in large cities.

Houssiau et al., 2023, Patterns *4*, 100662
January 13, 2023 © 2022 The Author(s).
https://doi.org/10.1016/j.patter.2022.100662

CellPress

Patterns

CellPress
OPEN ACCESS

Article

Detrimental network effects in privacy: A graph-theoretic model for node-based intrusions

Florimond Houssiau,[1,2,5] Piotr Sapieżyński,[3,5] Laura Radaelli,[4,5] Erez Shmueli,[4] and Yves-Alexandre de Montjoye[2,6,*]
[1]The Alan Turing Institute, 2QR, John Dodson House, 96 Euston Road, London NW1 2DB, UK
[2]Imperial College London, Exhibition Road, South Kensington, London SW7 2BX, UK
[3]Northeastern University, 360 Huntington Avenue, Boston, MA 02115, USA
[4]Department of Industrial Engineering, Tel Aviv University, P.O. Box 39040, Tel Aviv 69978, Israel
[5]These authors contributed equally
[6]Lead contact
*Correspondence: demontjoye@imperial.ac.uk
https://doi.org/10.1016/j.patter.2022.100662

THE BIGGER PICTURE Proportionality, ensuring that the data collected are relevant for the purposes of the processing is a key tenet of modern data protection laws, such as the EU General Data Protection Regulation. Evaluating proportionality when "small-scale" data are collected can already be difficult. This only becomes harder when entering the realm of "big data" and, in particular, (big) networked data. Indeed, a lot of data collected today intrinsically relate to more than one person. This includes social network data, messaging data, and close proximity data. From a data protection perspective, this means that even though data about only a handful of people are collected, information about many more people might be included in the dataset. This is what happened with Cambridge Analytica: through 270,000 accounts they collected data about 68.0M people. So far, we have not had a tool to estimate the number of people affected by networked data collection. We propose and validate such a tool.

1 2 3 4 5 **Proof-of-Concept:** Data science output has been formulated, implemented, and tested for one domain/problem

SUMMARY

Despite proportionality being one of the tenets of data protection laws, we currently lack a robust analytical framework to evaluate the reach of modern data collections and the network effects at play. Here, we propose a graph-theoretic model and notions of node- and edge-observability to quantify the reach of networked data collections. We first prove closed-form expressions for our metrics and quantify the impact of the graph's structure on observability. Second, using our model, we quantify how (1) from 270,000 compromised accounts, Cambridge Analytica collected 68.0M Facebook profiles; (2) from surveilling 0.01% of the nodes in a mobile phone network, a law enforcement agency could observe 18.6% of all communications; and (3) an app installed on 1% of smartphones could monitor the location of half of the London population through close proximity tracing. Better quantifying the reach of data collection mechanisms is essential to evaluate their proportionality.

INTRODUCTION

For reasons ranging from the democratization of communication technologies[1] to urbanization and rural exodus, we are today more connected than ever. The fraction of the population living in cities is increasing and projected to reach 68% by 2050,[2] while the average worldwide degree of separation dramatically shrank from 6 steps in 1969 to 3.5 steps today.[3–6] Many positive network effects arise from such connectedness, including increased average GDP[7,8] and number of patents produced per capita,[9] and an accelerated diffusion of information.[10,11] Network science emerged as a field to study human and societies through the lens of our connections.[12]

This connectedness, however, impacts our privacy as, in modern systems, (1) data often relate to more than one person (e.g., a text sent between two people) and (2) people often collect or have access to data about their friends and people around them (e.g., bluetooth close-proximity data). In both cases, the

This is an open access article under the CC BY license (http://creativecommons.org/licenses/by/4.0/).

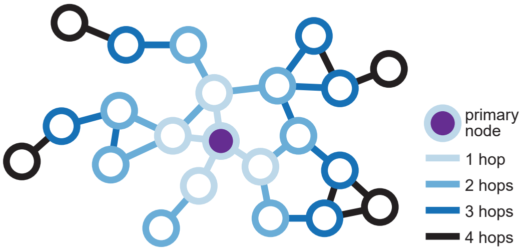

Figure 1. Node- and edge-observability in a network with one primary node

The direct neighbors (light blue) of the primary node, and the edges between the primary node and its neighbors are observable. The edges between the light blue nodes are, however, not observable. In the 2-hop case, nodes that are neighbors of nodes neighboring the primary node become observable. Similarly, all edges incident to a neighbor of the primary node become observable.

network effects associated with modern connectedness often strongly amplify the scope and privacy impact of data collections. Both the Snowden revelations[13] and Cambridge Analytica[14] leveraged these network effects. Yet, despite these being two of the major privacy scandals of the last decade, we currently still lack the analytical tools to correctly assess the reach and therefore proportionality of modern data collections.

Proportionality of data collections is one the tenets of data protection laws, such as the European Union's General Data Protection Regulation (GDPR) and Law Enforcement Directive (LED), and is an integral part of Privacy Impact Assessments (PIAs). The European Data Protection Supervisor (EDPS), for instance, states in its recent "Guidelines on assessing the proportionality of measures that limit the fundamental rights to privacy and to the protection of personal data" that to evaluate the proportionality of data collections one must assess both the "number of people affected" and whether it "raises 'collateral intrusions,' that is interference with the privacy of persons other than the subjects of the measure."[15] The importance of proportionality was further emphasized in the context of data collections by law enforcement agencies in the Opinion 01/2014 of the Article 29 Working Party, which emphasize the need for "the scope of the proposed measure [to be] sufficiently limited" in particular with respect to the "number of people affected by the measure."

Here, we propose a graph-theoretic model to evaluate the reach and therefore proportionality of modern data collections and attacks. We define a general model to measure the privacy loss for individuals when nodes in the network (the "primary" nodes) have their data, and data from their neighborhood, collected. Such data collections include installing a malicious app, having loose permissions on Facebook, and surrendering one's phone data to the authorities. We then define two metrics for the vulnerability of networks to these attacks, node- and edge-*observability*, reflecting what an attacker can learn. We prove closed-form expressions for the observability of networks. Finally, we show that (1) our model allows us to correctly estimate the number of Facebook accounts observed by Cambridge Analytica in 2014, using only the degree distribution of the graph; (2) under current legislation, surveillance of 0.01% random phones in a mobile phone network would allow a law enforcement agency to tap 18.6% of all communications occurring on that network; and (3) a software installed on 1% of the smartphones in London would enable its developer to monitor the hourly location of half of the population through close-proximity tracing.

RESULTS

We consider an undirected graph $G = (V,E)$, where V is the set of nodes and E is the set of undirected edges, i.e., unordered pairs $(u,v) \in E$ with $u,v \in V$. The edge-set of node u, the set of all its incident edges, is denoted by $E[u]$. We define $V_p \subset V$, the set of *primary nodes*, as the set that an attacker has gained control of through a node-based data collection or attack, e.g., a malicious app on Facebook or on a mobile phone. We denote this set's size by n_p. This model applies to both attacks and legitimate data collections, and we use these words interchangeably. An attacker can access all communication between any primary node $v \in V_p$ and its neighbors $\{u | (u,v) \in E[u]\}$ in the network (phone calls, private messages, …), as well as any attributes of v's neighbors that are available to v (name, location, profile, …). This results in a set of observed edges E_o and a set of observed nodes V_o. In some scenarios, the adversary may also observe nodes and edges located up to k hops away from the primary node (see Figure 1).

Definition 1. Observed nodes. The set of observed nodes after k hops, V_o^k, for a set V_p of primary nodes, contains all non-primary nodes that are at a shortest-path distance (noted d) at most k from a primary node (where d denotes the shortest path distance):

$$V_o^k \overset{def}{=} \{u \in V \backslash V_p : \exists v \in V_p, d(u,v) \le k\}$$

Definition 2. Observed edges. The set of observed edges after k hops E_o^k, for a set V_p of primary nodes, contains all edges that are connected to at least one node within shortest-path distance $k - 1$ of a primary node:

$$E_o^k \overset{def}{=} \{(u,v) \in E : \exists w \in V_p, d(u,w) \le k - 1 \vee d(v,w) \le k - 1\}$$

For simplicity, we also define: $V_o \overset{def}{=} V_o^1$ and $E_o \overset{def}{=} E_o^1$.

We then define two quantities to measure the reach of an attack, which we call edge-observability and node-observability. Edge-observability quantifies the amount of information transmitted between nodes accessible to an attacker; while node-observability quantifies the information about node attributes that this attacker has access to. We also differentiate between two different risks in both attack models: the privacy loss caused by an attack on the whole network, $\rho_\circ^{(k)}(G,n_p)$, and by attacks on one particular node u, $\rho_{n,*}^{(k)}(G,n_p,u)$, or edge e, $\rho_{e,*}^{(k)}(G,n_p,e)$.

Finally, we focus here on non-targeted node-based intrusions as, in many real-world scenarios, including the ones we consider here, such data collections or attacks are opportunistic rather than targeted: people install an app, click on a phishing link and enter their password, or pass by a malicious router in the street. In both attacks, the set V_p is thus chosen uniformly at random: the probability for a node to be part of the data collection is uniform. Non-random attacks, when appropriate, can, however, be empirically studied using the same model. We discuss this in supplemental information section S1.

CellPress
OPEN ACCESS

As all of our metrics depend on the number of primary nodes n_p, we also introduce the average node- and edge-observability, defined as $\frac{1}{N}\sum_{n_p=1}^{N} f(G,n_p)$ for a metric f. This number summarizes the observability curve and can be used as a measure of how vulnerable the network is to node-intrusion attacks in general.

Node-observability

The node-observability of a graph quantifies an attacker's ability to obtain knowledge about the attributes of a node through their relationship with primary nodes. Such attacks could, for instance, be conducted by malicious apps on social networks exploiting loose permissions to obtain the installer's friends information.[16]

Definition 3. Probability of observation of a node. The $k-$ hop probability of observation of a node u, in a graph $G = (V,E)$, for n_p primary nodes, $\rho_{n,*}^{(k)}(G,n_p,u)$, is defined as the probability that u is observed, with the probability taken over all sets V_p of size n_p such that $u \notin V_p$:

$$\rho_{n,*}^{(k)}(G,n_p,u) = \mathbb{P}_{\sim V_p}\left[u \in V_o^k \;\middle|\; u \notin V_p \wedge |V_p| = n_p\right]$$

Definition 4. Node-observability of a graph. The $k-$ hop node-observability of a graph $G=(V,E)$ for n_p primary nodes, $\rho_n^{(k)}(G,n_p)$, is defined as the average fraction of nodes an attacker can observe, where the expectation is taken over all sets V_p of size n_p:

$$\rho_n^{(k)}(G,n_p) = \mathbb{E}_{\sim V_p}\left[\frac{|V_p| + |V_o^k|}{|V|}\middle| |V_p| = n_p\right]$$

These two quantities are linked one to another, as shown in theorem 1.

Theorem 1. Let $G=(V,E)$ be a graph with $n=|V|$ nodes, and k a positive integer. The $k-$ hop node-observability of G for n_p primary nodes is equal to:

$$\rho_n^{(k)}(G,n_p) = \frac{n_n}{n} + \frac{n-n_p}{n}\cdot\frac{1}{n}\sum_{u\in G}\rho_{n,*}^{(k)}(G,n_p,u)$$

Theorems 2 and 5, which we propose and prove, give provable closed-form expressions for the probability of observation of a node and the node-observability of a graph. These theorems rely on the $k-$ hop degree of a node, i.e., the number of other nodes that are within at most k-hops from that node. (In particular, note that the 1-hop degree of a node is its degree.) In the 1-hop case, these expressions can be used to estimate node-observability and probability of observation, using only the full degree list of the graph. This quantity has been extensively studied and reported in the network science literature (see, e.g., Barabasi and Albert[17] and Watts and Strogatz[18]).

Theorem 2. Let $G=(V,E)$ be a graph with $n=|V|$ nodes, and $n_p \le n$ a number of primary nodes. Let $u\in V$ be a node, the $k-$ hop probability of observation of u is given by:

$$\rho_{n,*}^{(k)}(G,n_p,u) = \begin{cases} 1 - \dfrac{C_{n_p}^{n-1-deg_k(u)}}{C_{n_p}^{n-1}} & \text{if } deg_k(u) \le n-1-n_p \\ 1 & \text{otherwise.}\end{cases}$$

Where $\deg_k(u)$ is the $k-$ hop degree:

$$\deg_k(u) = |\{v \in V\backslash\{u\} : d(u,v) \le k\}|$$

Theorem 3. Let $G=(V,E)$ be a graph with $n=|V|$ nodes, and $n_p \le n$ a number of primary nodes. Let $d\in\mathbb{N}^n$ be the distribution of $k-$ hop degrees, defined as $d_i = |\{u \in V : \deg_k(u) = i-1\}|$. The $k-$ hop node-observability of G is given by:

$$\rho_n^{(k)}(G,n_p) = \frac{n_p}{n} + \frac{n-n_p}{n}\sum_{i=1}^{n} d_i f(n_p, i-1)$$

Where f is defined as:

$$f(n_p,d) = \begin{cases} 1 - \dfrac{C_{n_p}^{n-1-d}}{C_{n_p}^{n-1}} & \text{if } d \le n-1-n_p \\ 1 & \text{otherwise.}\end{cases}$$

Edge-observability

The edge-observability of a graph quantifies the attacker's ability to obtain edge-based information happening in the network when the data collector has access to n_p random primary nodes. Edge-observability can, for instance, be used to model the ability of a government agency to surveil communications occurring on a phone network.

Definition 5. Probability of observation of an edge. The $k-$ hop probability of observation of an edge $e = (u,v)$, in a graph G, for n_p primary nodes, $\rho_{e,*}^{(k)}(G,n_p,e)$, is defined as the probability that e is observed, with the probability taken over all sets V_p of size n_p:

$$\rho_{e,*}^{(k)}(G,n_p,e) = \mathbb{P}_{\sim V_p}\left[e \in E_o^k \middle| |V_p| = n_p\right]$$

Definition 6. Edge-observability of a graph. The $k-$ hop edge-observability of a graph G for n_p primary nodes, $\rho_e^{(k)}(G,n_p)$, is defined as the expected fraction of the edge-set of a node u that the attacker can observe, where the expectation is taken over all sets V_p of size n_p:

$$\rho_e^{(k)}(G,n_p) = \mathbb{E}_{\sim V_p}\left[\frac{|E_o^k|}{|E|}\middle| |V_p| = n_p\right]$$

In theorems 4 and 5, we prove closed-form expressions to estimate the edge-observability of a graph and the probability of an observation of an edge in the k-hop case, using the notion of $k-$ hop edge-degree, the number of nodes within distance $k-1$ of either extremity of the edge. Importantly, we furthermore show that the average edge-observability can be computed from the number of nodes only (in the 1-hop case).

Theorem 4. Let $G=(V,E)$ be a graph with N nodes, and $n_p \le N$ a number of primary nodes. The $k-$ hop edge-observability of G is equal to the average $k-$ hop probability of observation of edges $e\in E$.

$$\rho_e^{(k)}(G,n_p) = \frac{1}{|E|}\sum_{e\in E}\rho_{e,*}^{(k)}(G,n_p,e)$$

Theorem 5. Let $G=(V,E)$ be a graph with $n=|V|$ nodes, and $n_p \le n$ a number of primary nodes. Let $e=(u,v)\in E$ be

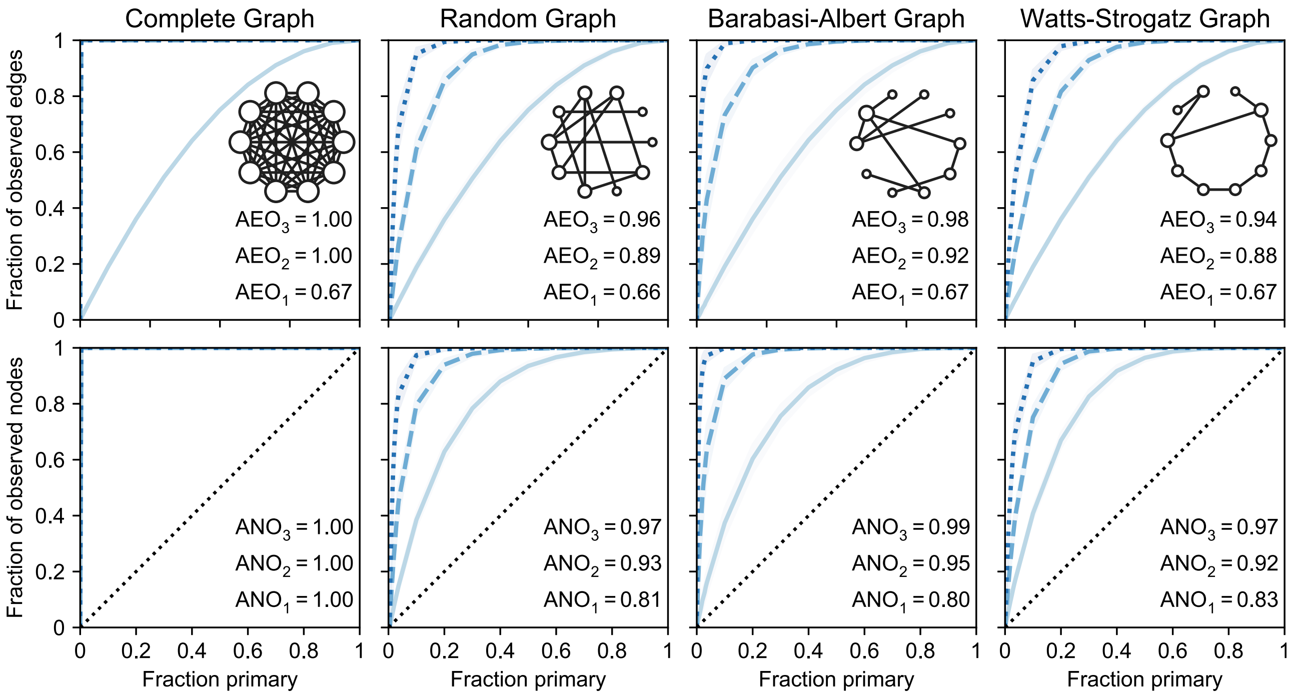

Figure 2. 1-hop (light blue), 2-hop (dashed, blue) and 3-hop (dotted, dark blue) edge- and node-observability of synthetic graphs as a function of the fraction of primary nodes

For each graph, we report the average node-observability (ANO) and average edge-observability (AEO). Shaded area, 1 ± SD around the mean.

an edge. The $k-$ hop probability of observation of e is given by:

$$\rho_{e,*}^{(k)}(G,n_p,e) = \begin{cases} 1 - \dfrac{C_{n_p}^{n-edgedeg_k(e)}}{C_{n_p}^{n}} & \text{if } edgedeg_k(e) \le n - n_p \\ 1 & \text{otherwise} \end{cases}$$

Where $edgedeg_k(e)$, for $e = (u,v)$, is the $k-$ hop edge-degree of e:

$$edgedeg_k(e) = |\{w \in V : d(u,w) \le k-1 \vee d(v,w) \le k-1\}|$$

Corollary 1. Let $G = (V,E)$ be a graph with $n = |V|$ nodes, and $n_p \le n$ a number of primary nodes. Let $e = (u,v) \in E$ be an edge. The $1-$ hop probability of observation of e is identical for all edges, and given by:

$$\rho_{e,*}^{(1)}(G,n_p,e) = 1 - \frac{n-n_p}{n} \cdot \frac{n-n_p-1}{n-1}$$

Impact of graph structure

In this section, we empirically investigate how the structure of the graph impacts its vulnerability to node-based data collections in the 2- and 3-hop cases. We measure observability for four families of synthetic graphs: complete graphs, Erdős-Rényi,[19] Barabási-Albert,[17] and Watts-Strogatz[18] graphs. Each graph is generated with a comparable number of nodes and edges (except for the complete graph, which always has $|E| = \frac{|V|(|V|-1)}{2}$), but exhibit different structure (see "empirical study of observability").

First, we validate our theoretical results for 1-hop. We show in Figure 2 that (1) the 1-hop edge-observability is identical for all types of graphs, as expected from theorems 4 and 5; (2) the node-observability and the average probability of observation of a node are near identical for all number of hops and small values of n_p, as expected from theorem 1. Figure 3 shows that the 1-hop average edge-observability is constant at $2/3$ for increasing graph density, a direct consequence of theorem 1. It further displays the sharp increase of the 1-hop average edge-observability with density.

Second, Figure 2 shows the impact of the structure of a graph on its 2- and 3-hop node- and edge-observability. More specifically, Barabási-Albert graphs have larger average observability (for all metrics) than Erdős-Rényi graphs, which in turn have slightly higher average observability than Watts-Strogatz graphs. This is likely due to Barabási-Albert graphs containing, by design, very high-degree nodes called hubs. Their high degree makes hubs more likely to be observed which, in turn, allows an attacker to observe their neighbors (2-hop) and the neighbors of their neighbors (3-hop), thereby strongly increasing the observability of the graph. Watts-Strogatz graphs, on the other hand, present a lattice-like structure with high clustering coefficient.[18] Observing data from primary nodes is therefore likely to have a fairly local impact on observability, as opposed to spanning over the entire graph, hence decreasing the node- and edge-observability of the graph.

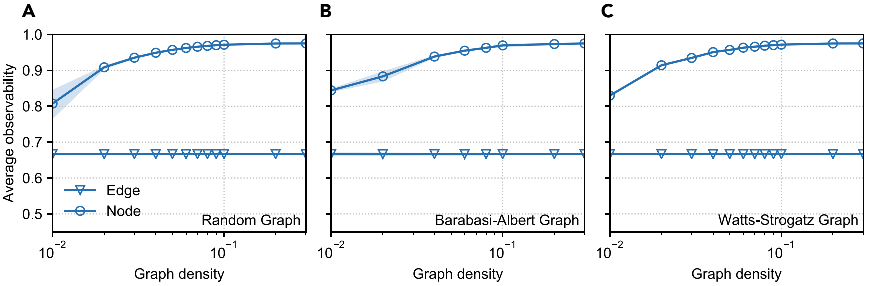

Figure 3. Average 1-hop edge- and node-observability of graphs with increasing density
Erdős-Rényi (A), Barabási-Albert (B), and Watts-Strogatz (C) graphs. The 1-hop average edge-observability is constant (thm 5), while the 1-hop average node-observability increases sharply with density. Barabási-Albert graphs tend to be more observable than others, probably due to the presence of hubs. Shaded area contains all data points except the top and bottom 5%.

Social networks

In 2018, it was revealed that Cambridge Analytica, a UK company, had obtained data from about 70M US Facebook profiles through an innocuous-looking app. Once installed, the app would collect the data of the person who installed it as well as information about their Facebook friends (1-hop).[20,21] This led to investigations by several governments, including the UK and the US, and resulted in Facebook receiving a record $5 billion fine from the FTC.[22] This was, to the best of our knowledge, the first major instance of a node-observability attack based on node-intrusions on social networks.

Using our model, theorem 2 and the shifted degree distribution of Facebook users in the US,[4] we were able to quantify the 1-hop node-observability of the Facebook network. Figure 4B shows that, for the reported 270,000 app installs, the attacker should have been able to observe 68.0M profiles ($\rho_n^{(1)}(G,\ n_p)\ =\ 0.318$). This contradicts initial reports that data about 50M people had been breached, and is very close to later reports by Facebook that 70.6M US accounts had been breached.[21] Similarly, our results show that, with 1M reported app installs, the Obama campaign might have had access to data of up to 94.8M people ($\rho_n^{(1)}(G, n_p)\ =\ 0.510$).

Theorem 5 furthermore allows us to derive $\rho_{n,*}^{(1)}(G, n_p, u)$, the probability of a person to have had their data collected, as a function of their degree $\deg(u)$. Figure 4A shows that an average user had a 36% chance of having their profile collected by Cambridge Analytica and 79% by the Obama Campaign. Users with as little as 100 Facebook friends would have had a 12.3% (respectively 41.7%) risk of having their profile collected by Cambridge Analytica (resptively, the Obama campaign), while these numbers increase to 48.2% (respectively, 93.2%) for users with 500 friends.

Finally, we evaluate the effectiveness of these attacks under a 2-hop policy, i.e., allowing apps to collect data on not only the friends, but also the friends of friends, of accounts installing them. Using a synthetic Facebook graph based on the configuration model[23] and the known degree distribution, we show that a 2-hop policy would have allowed both Cambridge Analytica and the Obama campaign to observe virtually every single US profile. We provide more details on our experimental methodology in the section S2.

Note that, although the error we observe between the node-observability of the model and the value published is relatively low (3.7%), this is only one data point. Our estimator has two main sources of error: (1) the estimation of the degree distribution from 2011 data, and (2) whether our assumption that primary nodes are sampled uniformly at random holds. Further work is needed to quantify the impact the different sources of error have on the accuracy of the model. This is, however, challenging as an empirical analysis of error (2) would require data from many real-world attacks.

Mobile phone networks

Surveillance of mobile phone networks was one of the first and most significant revelations by whistleblower Edward Snowden in 2013. The list of who we talked to on the phone—the edges of our social graph—is sensitive and considered private by 63% of Americans.[24] Recognizing that this information might help fight crimes and terrorism, legislation allowing law enforcement to access a suspect's mobile phone records have been enacted in the past two decades around the world.[25,26] Several of these legislations, including in the US, also allow agencies to collect the mobile phone records (edges) of people up to two hops from the suspects (contacts of contacts), effectively recording communications occurring along 3-hop edges. For instance, in an attempt to curb illegal immigration, the US Border Patrol was allowed to use call logs to confirm the legal status of their potential target and to search for other potential illegal immigrants among the target's contacts.[27] Similarly, the USA PATRIOT act allowed intelligence agencies to collect phone

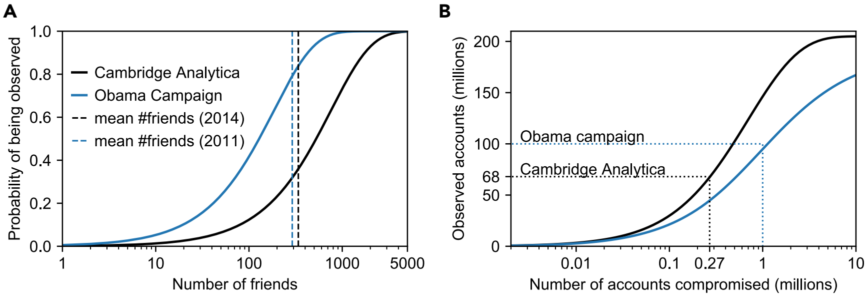

Figure 4. Node-observability of the Facebook network in 2014
(A) Probability of a node being observed by Cambridge Analytica or the Obama campaign as a function of the node's degree. The vertical dashed line is the average number of friends in 2014.
(B) Node-observability (in absolute numbers) of the network as a function of the number of primary nodes (accounts installing the app), for 1- and 2-hops.

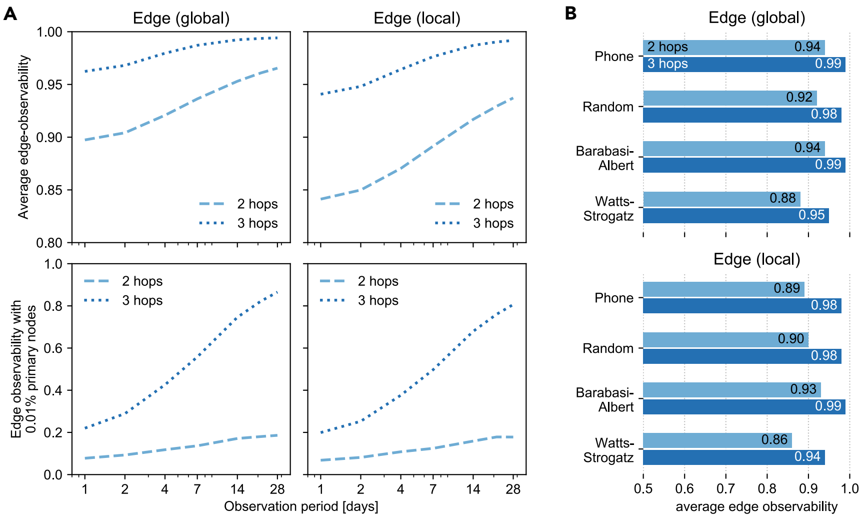

Figure 5. 2-hop and 3-hop edge-observability of a mobile phone network

(A) 2- and 3-hop average edge-observability (top), and edge-observability when 0.01% of the nodes are primary (bottom), of the real phone network as the observation window increases (in log-scale).

(B) Comparison of the average edge-observability of the real phone network (observation window of 7 days) and synthetic networks with the same number of nodes and edges.

records of people up to three hops from suspects. This number was reduced to two hops in 2015, following the Snowden revelations.[28]

Here, we use a real-world mobile phone dataset of 1.4M people over 1 month to study and quantify the potential of node-based data collections for surveillance. In this attack, we consider two nodes to be connected to one another if they interacted (call or text) at least once in the time period studied.

Figure 5A shows that, under the previous 3-hop policy, collecting data from 0.01% of the population would allow an attacker to surveil 86.6% of all the communications happening in the network (edge-observability of the graph). While the new 2-hop policy decreases those numbers, we found that it still allows an attacker to surveil 18.6% of all the communications. Finally, we show that the average edge-observability, when 1% of the network is primary, (1) increases sharply with the length of the observation period and (2) already reaches 53.3% when observing only one day of mobile phone metadata in the 3-hop case.

Figure 5B shows that the edge-observability of the phone network (7-day observation period) is comparable with that of a Barabási-Albert graph with similar density and number of nodes. As our previous results show, this is likely due to the presence of hubs in the mobile phone network. Figure 6 shows that both graphs have a similar degree distribution, including hubs, as is expected from previous research.[29]

Close proximity networks

Mobility data are considered to be among the most sensitive data currently being collected.[24] Here, we use real-world Bluetooth close proximity data to investigate the feasibility and reach of a distributed node-observability attack through proximity sensing by compromised apps (e.g., a fake "flashlight"[30]) or contact tracing apps. In this attack, an attacker monitors the location of nearby uncompromised devices using the GPS location and sensing capabilities of compromised (primary) phones.

We empirically estimate the node-observability of this attack on a co-location dataset of 600 people collected as part of the Copenhagen Networks Study in 2014.[31] We extract hourly co-location graphs of phones who sensed each other within that hour, for different time periods and spatial regions (all of 1 km^2, see Figure 8A). From these hourly graphs, we compute the average probability of observation of a node as a function of the number of primary nodes per km^2, and fit an approximation curve $\hat{\mu}(n_p) = \min(1, \max(0, A\log(n_p) + B))$ ($R^2 = 0.876$). From publicly available census data, we extract the population count in each 1 km^2 cell. We then combine these counts with $\hat{\mu}(\circ)$ to estimate the node-observability of large cities (see "observability of cities" and section S3 for more details). In Figure 7, we compare the network properties of two hourly graphs (one during daytime and the other during nighttime) with graph models. We show that the nighttime graph is well approximated by a Barabási-Albert graph, while the daytime graph has a bi-modal degree distribution that is not well approximated by the graph models we consider.

Figure 8B shows that, using this attack in London, an attacker might be able to observe the location of as much as 57% of individuals (resp. 86%) with only 1% (resp. 10%) of the population installing the app, giving an average node-observability of 0.95. Other dense cities we considered—Chicago, Los Angeles, and Singapore—display a similarly large (>0.876) average node-observability.

Conversely, the attack is a lot less effective in sparsely populated areas where the average degree of the close proximity networks is lower. For instance, we estimate that in Utah (15 people per km^2 on average, while London has 4,500) an app installed by 1% (resp. 10%) of the population would allow an attack to observe only 9% (resp. 38%) of individuals in the state, an average node-observability of 0.70.

Although the dataset we use is from 2014, we believe that our results still apply to modern datasets. Indeed, we do not expect human mobility to have changed significantly (outside of the COVID-19 pandemic) between 2014 and now. In the discussion, we further discuss how mobile phone applications could be leveraged to perform a similar attack.

DISCUSSION

Previous quantitative works have explored the impact of groups on individual privacy through the privacy loss incurred by homophily, the similarity between connected individuals in social networks[32] as the right of individuals to keep their affiliation with a group[33–35]—a religion, a sexual orientation, a disease, etc.—private. Researchers have then developed technical solutions to

A

network type	avg. clustering	modularity
Phone	1.6×10^{-1}	0.72
Random	3.7×10^{-6}	0.43
Barabasi-Albert	1.0×10^{-4}	0.36
Watts-Strogatz	2.7×10^{-1}	0.81

B

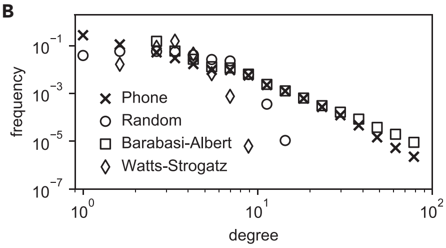

Figure 6. Network properties of our phone network and three synthetic graphs

(A) Modularity and average clustering coefficient for the mobile phone network and three synthetic graphs with identical number of nodes and density. Watts-Strogatz's resulting average clustering coefficient is the closest one to that of our phone network.

(B) Degree distribution for the mobile phone dataset network and the synthetic graph models with equal number of nodes and density. The degree distribution of a Barabási-Albert graph appears to be the closest one to the empirical distribution.

protect this affiliation (such as t-closeness[36] or group-based obfuscation[37]).

Extensive prior work also studied the robustness of networks to node removal.[38] Similarly to our work, nodes (and potentially their $l-$ hop neighborhood[39]) are compromised. However, the focus of such work is on estimating the impact of compromised node on the connectivity of the graph and its percolation clusters. Our work here focuses on the observability of graphs and its impact on privacy.

In our mobile phone network experiment, we assumed that the data collector could gain access to a large number of primary nodes. In practice, surveillance in phone networks is happening in numerous countries, sometimes at large scale. For instance, ANTENJ, a French national agency dedicated to "numerical judiciary investigations" has over 10,000 phone numbers under surveillance at any point in time.[26] To the best of our knowledge, the only quantitative study of surveillance of phone networks has been conducted by Mayer et al.[28] It concluded that hubs increase the number of nodes that can be reached through 3 hops. Their analysis is, however, entirely based on a disconnected dataset of less than 1,000 users collected through an app. The small size of the dataset and the inherent sampling bias prevented them from (1) quantifying the potential of node-based intrusions for mass and targeted surveillance, (2) comparing their results to graph models, and (3) showing the importance of the graph's clustering coefficient in edge-observability.

In close proximity tracking attacks, phones could for instance be compromised by malware, code embedded by third-parties, or legitimate applications, such as contact tracing apps. The Judy malware was for instance estimated to have infected 36M devices worldwide,[40] while a fake flashlight app was discovered to collect data from tens of millions of users.[41] The UK start-up Tamoco, which reported having deals to embed their code in 1,000 Android apps, giving them access to 100M devices,[42] while the software of US company SignalFrame uses WiFi to scan for nearby devices and record their location.[43] Similarly, Pegasus, a spyware developed by NSO, has been employed to compromise the devices of targeted journalists, politicians, and activists in multiple countries.[44] Finally, contact tracing applications, such as those designed to measure the spread of COVID-19, are designed to track co-location of neighboring phones[45] by having phones broadcast a unique identifier. If identifiers are consistent or linkable across time, they could be used for large-scale co-location tracing.

Close proximity data can be collected through Wi-Fi or Bluetooth. Attacks based on Wi-Fi are either passive, where the app simply observes probe requests that mobile phones send to sense nearby Wi-Fi hotspots, or active, running a fake hotspot with a common SSID (e.g., `attwifi`, `xfinitywifi`) for nearby phones to connect to.[46] While more complex, the latter bypasses MAC address randomization used by some operating systems, including relatively recent versions of both Android and iOS.[47,48] The two main platforms are thus moving toward re-randomization of the MAC address even when connecting to previously known networks.[49] These changes will, however, take years to reach all smartphone users. Attacks based on Bluetooth, originally considered less likely, might be enabled by

A

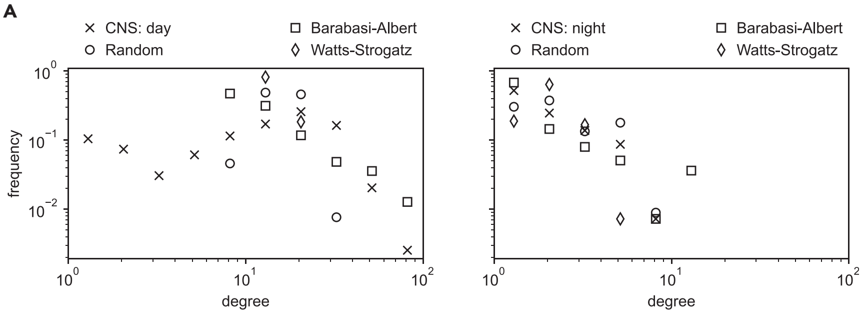

B

network type	avg. clustering	modularity
CNS: day	4.9×10^{-1}	0.55
Random	3.9×10^{-2}	0.22
Barabasi-Albert	1.0×10^{-1}	0.22
Watts-Strogatz	3.7×10^{-1}	0.52

network type	avg. clustering	modularity
CNS: night	7.7×10^{-1}	-
Random	2.4×10^{-1}	0.72
Barabasi-Albert	-	0.81
Watts-Strogatz	-	0.83

Figure 7. Network properties of daytime and nighttime close proximity networks

(A) Degree distribution for the Copenhagen Networks Study networks (a day snapshot and a night snapshot) and the synthetic graph models with equal number of nodes and density. The day snapshot has a bimodal degree distribution that is not well reflected by any synthetic network. The night snapshot has a degree distribution similar to a Barabasi-Albert network.

(B) Modularity and average clustering coefficient for Copenhagen Networks Study networks and three synthetic graphs with identical number of nodes and density. Watts-Strogatz's resulting average clustering coefficient is the closest one to day snapshot of CNS. The night snapshot is too sparse to meaningfully calculate clustering and modularity. Overall, the considered graph models are not a good fit for close proximity networks.

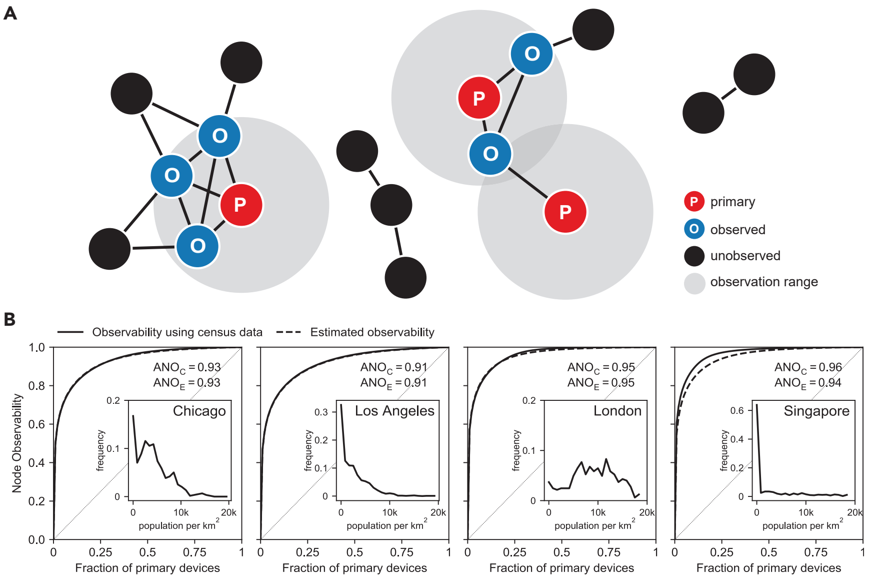

Figure 8. Observability of a co-location network and estimation of the node-observability of cities

(A) Compromised devices sense the presence of other devices within the observation range. This creates, for each time period, a co-location graph on which we compute local and global node observability.

(B) Node-observability in cities using exact census data (ANO_C) and estimation by an exponential distribution (ANO_E). Inset: distribution of the population per block of 1 km^2 of each city.

recent uses of Bluetooth for contact tracing apps against COVID-19.[45] Notably, the use of frequent Bluetooth MAC address and broadcast payload randomization in these apps lowers the privacy threat. A motivated attacker may, however, still track either very partial location histories or recent histories of the users who report themselves COVID-positive.[50,51]

Close proximity sensing also presents an additional risk: primary users also reveal which other users they interact with, i.e., the edges of their close proximity network along with potentially other information, such as the time of the interaction or distance between the two phones. This risk has been heavily discussed in the case of contact tracing apps relying on the so-called centralized model.[51] Corollary 1 shows that the resulting edge-observability (fraction of the edges observed) is independent from the graph's structure, being equal to $1 - \frac{n-n_c}{n} \cdot \frac{n-n_c-1}{n-1} \approx 1 - \left(1 - \frac{n_c}{n}\right)^2$. This means that a contact tracing app that would report 15 days of a user's interactions to a server once that user is diagnosed positive would reveal, on average, 2.0% of the 15 days social graph to the server if 1% of the population is infected; and 18.9% if 10% of the population is infected. While network effects are still at play, they are a lot less strong than in the edge-observability case.

Table 1. Node-observability $\rho_n^{(1)}(G, n_p)$ for the 2014 Facebook graph and $n_p = 270,000$ accounts observed when the maximum degree is capped

Degree cap	Fraction of nodes affected (%)	Observability
5,000	0.00	0.329
4,000	0.03	0.329
2,000	0.29	0.329
1,000	2.94	0.326
500	17.21	0.305
250	49.23	0.243

The observability only decreases slightly with the cap, except when a large fraction of nodes are affected.

The attacks we model exploit the structure and connectedness of networks. We therefore believe the main mitigation to be to limit access to the networks as described earlier in this section. Another potential approach would be to modify the network in order to limit its observability. Here, we study whether capping the maximum degree that nodes can have in a network reduces the risk. We apply this cap to the social network setup (see results), starting from 5,000 (the maximum degree of that graph). We repeat our 1-hop analysis, measuring node-observability for $n_p = 270,000$, with a capped degree distribution $\widehat{P}_\kappa$ obtained by counting all nodes with degree higher than κ as nodes with degree κ $(\widehat{P}_\kappa(\kappa) = \sum_{i=\kappa}^{5000} \widehat{P}_\kappa(i)$ and $\widehat{P}_\kappa(i) = 0, \forall i > \kappa)$. We show in Table 1 that, unless the cap is large enough to affect a significant fraction of all users $(>20\%)$, the node-observability of the network remains largely unchanged. Protecting users from attacks by capping the maximum degree of nodes thus comes at a steep cost in utility, which might not be acceptable in practice.

This work is, to the best of our knowledge, the first to formalize and develop graph-theoretic models to understand the reach and therefore proportionality of modern data collection. Our results shed light on how network effects can have a strong detrimental impact on our privacy. The reliance on network effects to collect data by Cambridge Analytica is one of the first large-scale examples of a node-based data collection and is unlikely to be the last. We believe models like the one we present here to be essential to reason about data collection in networked environments. Moving forward, we hope this work can help evaluate the scope of data collections mechanisms and technologies and ensure their proportionality.

EXPERIMENTAL PROCEDURES

Resource availability

Lead contact

Further information and requests for resources should be directed to and will be fulfilled by the lead contact, Yves-Alexandre de Montjoye (deMontjoye@imperial.ac.uk).

Materials availability

There are no physical materials associated with this study.

Data and code availability

Code to compute both edge and node observability and data will be made available at https://github.com/computationalprivacy/network-privacy.

The mobile phone data cannot be made available for privacy and confidentiality reasons. Instead, we will make available synthetic data for the mobile phone graph using the Barabási-Albert graph model. The social network data are available from Ugander et al.[4] The Copenhagen dataset is available at Sapieżyński et al.[52] Finally, we will make the population and area data about cities available.

Proofs

In order to prove the theorems in the paper, we introduce a useful lemma.

Lemma 1. Let $G = (V, E)$ be a graph with $n = |V|$ nodes, $n_p \leq N$ a number of primary nodes, and $S \subset V$. The following holds when V_p is drawn uniformly at random:

$$\mathbb{P}_{\sim V_p \subset V}[\exists u \in V_p : u \in S| \, |V_p| = n_p] = \begin{cases} 1 - \dfrac{C_{n_p}^{n-|S|}}{C_{n_p}^{n}} & \text{if } |S| \leq n - n_p \\ 1 & \text{otherwise} \end{cases}$$

Proof. First, we rewrite the probability by considering its negation:

$$\begin{aligned} \mathbb{P}_{\sim V_p \subset V}[\exists u \in V_p : u \in S||V_p| = n_p] &= 1 - \mathbb{P}_{\sim V_p \subset V}[\forall u \in V_p : u \notin S||V_p = n_p] \\ &= 1 - \mathbb{P}_{\sim V_p \subset V}[V_p \cup S = \varnothing||V_p| = n_p] \\ &= 1 - \mathbb{P}_{\sim V_p \cup V}[V_p \subset V \backslash S||V_p| = n_p] \end{aligned}$$

Writing this probability in expectation of an indicator form yields:

$$\mathbb{P}_{\sim V_p \subset V}[V_p \subset V \backslash S||V_p| = n_p] = \sum_{v_p \subset V, |v_p| = n_p} \mathbb{P}[V_p = v_p] \cdot I\{v_p \subset V \backslash S\}$$

Since V_p is drawn uniformly at random, and a set of size n has $C_{n_p}^{n}$ subsets of size n_p, hence:

$$\mathbb{P}[V_p = v_p] = \frac{1}{|\{v_p \subset V : |v_p| = n_p\}|}$$

Furthermore, we have that, by definition:

$$\sum_{v_p \subset V, |v_p| = n_p} I\{v_p \subset V \backslash S\} = |\{v_p \subset V \backslash S : |v_p| = n_p\}|$$

The number of subsets of size n_p of a set of size m is $C_{n_p}^{m}$. Also, since that $S \subset V$, $|V \backslash S| = |V| - |S|$, and hence we obtain:

$$\mathbb{P}_{\sim V_p \subset V}[V_p \subset V \backslash S||V_p| = n_p] = \frac{|\{v_p \subset V \backslash S : |v_p| = n_p\}|}{|\{v_p \subset V : |v_p| = n_p\}|} = \frac{C_{n_p}^{n-|S|}}{C_{n_p}^{n}}$$

Theorem 1. Let $G = (V, E)$ be a graph with $n = |V|$ nodes, and k a positive integer. The $k-$hop node-observability of G for n_p primary nodes is equal to:

$$\rho_n^{(k)}(G, n_p) = \frac{n_n}{n} + \frac{n - n_p}{n} \cdot \frac{1}{n} \sum_{u \in G} \rho_{n,*}^{(k)}(G, n_p, u)$$

Proof. By definition and linearity of the expectation, we have (for the sake of clarity, we omit the conditional on $|V_p| = n_p$ in expectations):

$$\rho_n^{(k)}(G, n_p) = \mathbb{E}_{\sim V_p}\left[\frac{|V_p| + |V_o^k|}{|V|}\right] = \frac{n_p}{n} + \frac{1}{n} \cdot \mathbb{E}_{\sim V_p}[|V_o^k|]$$

To compute the last term, we write $|V_o^k|$ as the sum of the indicator of whether a node is in V_o:

$$\mathbb{E}_{\sim V_p}[|V_o^k|] = \mathbb{E}_{\sim V_p}\left[\sum_{u \in G} I\{u \in V_o^k\}\right] = \sum_{u \in G} \mathbb{P}_{\sim V_p}[u \in V_o^k]$$

To relate $\mathbb{P}_{\sim V_p}[u \in V_o^k]$ to $\rho_{n,*}^{(k)}(G, n_p, u)$, we develop it conditionally to the event $u \in V_p$, as, for any node $u \in G$:

$$\begin{aligned} \mathbb{P}_{\sim V_p}[u \in V_o^k] = \; & \mathbb{P}_{\sim V_p}[u \in V_o^k | u \in V_p] \mathbb{P}_{\sim V_p}[u \in V_p] + \\ & \mathbb{P}_{\sim V_p}[u \in V_o^k | u \notin V_p] \mathbb{P}_{\sim V_p}[u \notin V_p] \end{aligned}$$

By definition, $V_p \cap V_o^k = \varnothing$, and thus the first term is zero. Since V_p is a set of n_p nodes selected uniformly at random from n nodes, $\mathbb{P}_{\sim V_p}[u \notin V_p] = \frac{n - n_p}{n}$. Hence:

$$\mathbb{P}_{\sim V_p}[u \in V_o^k] = \frac{n - n_p}{n} \cdot \rho_{n,*}^{(k)}(G, n_p, u)$$

Theorem 2. Let $G = (V, E)$ be a graph with $n = |V|$ nodes, and $n_p \leq n$ a number of primary nodes. Let $u \in V$ be a node, the $k-$hop probability of observation of u is given by:

$$\rho_{n,*}^{(k)}(G, n_p, u) = \begin{cases} 1 - \dfrac{C_{n_p}^{n-1-deg_k(u)}}{C_{n_p}^{n-1}} & \text{if } deg_k(u) \leq n - 1 - n_p \\ 1 & \text{otherwise.} \end{cases}$$

Where $\deg_k(u)$ is the $k-$hop degree:

$$\deg_k(u) = |\{v \in V \backslash \{u\} : d(u, v) \leq k\}|$$

Proof. First, from the definition of V_o, we obtain:

$$\begin{aligned} \rho_{n,*}^{(k)}(G, n_p, u) &= \mathbb{P}_{\sim V_p \subset V}[u \in V_o^k | u \notin V_p, |V_p| = n_p] \\ &= \mathbb{P}_{\sim V_p \subset V}[\exists v \in V_p : d(u, v) \leq k | u \notin V_p, |V_p| = n_p] \\ &= \mathbb{P}_{\sim V_p \subset V}[\exists v \in V_p : d(u, v) \leq k | V_p \subset V \backslash \{u\}, |V_p| = n_p] \\ &= \mathbb{P}_{\sim V_p \subset V}[\exists v \in V_p : v \in neigh_k(u) | V_p \subset V \backslash \{u\}, |V_p| = n_p] \end{aligned}$$

Where $neigh_k(u) = \{v \in V \backslash \{u\} : d(u, v) \leq k\}$. Note that, $|neigh_k(u)| = \deg_k(u)$.

Define $G' = (V', E') = (V \backslash \{u\}, E \backslash E[u])$, the graph without u. We can then write:

$$\rho_{n,*}^{(k)}(G, n_p, u) = \mathbb{P}_{\sim V_p \subset V'}[\exists v \in V_p : v \in neigh_k(u)||V_p| = n_p]$$

Since $neigh_k(u) \subset V'$, we can apply lemma 1 on G' with $S = neigh_k(u)$ (observe that $|V'| = n - 1$), which concludes the proof.

Theorem 3. Let $G = (V, E)$ be a graph with $n = |V|$ nodes, and $n_p \leq n$ a number of primary nodes. Let $d \in \mathbb{N}^n$ be the distribution of $k-$hop degrees, defined as $d_i = |\{u \in V : \deg_k(u) = i - 1\}|$. The $k-$hop node-observability of G is given by:

$$\rho_n^{(k)}(G, n_p) = \frac{n_p}{n} + \frac{n - n_p}{n} \sum_{i=1}^{n} d_i f(n_p, i - 1)$$

Where f is defined as:

$$f(n_p, d) = \begin{cases} 1 - \dfrac{C_{n_p}^{n-1-d}}{C_{n_p}^{n-1}} & \text{if } d \leq n - 1 - n_p \\ 1 & \text{otherwise.} \end{cases}$$

Proof. This follows immediately from theorems 1 and 2, using the fact that the probability of observation of a node depends only on its degree.

Theorem 4. Let $G = (V, E)$ be a graph with $n = |V|$ nodes, and $n_p \leq n$ a number of primary nodes. Let $d \in \mathbb{N}^n$ be the distribution of $k-$hop degrees, defined as $d_i = |\{u \in V : \deg_k(u) = i - 1\}|$. The $k-$hop node-observability of G is given by:

$$\rho_n^{(k)}(G, n_p) = \frac{n_p}{n} + \frac{n - n_p}{n} \sum_{i=1}^{n} d_i f(n_p, i - 1)$$

Where f is defined as:

$$f(n_p, d) = \begin{cases} 1 - \dfrac{C_{n_p}^{n-1-d}}{C_{n_p}^{n-1}} & \text{if } d \leq n - 1 - n_p \\ 1 & \text{otherwise.} \end{cases}$$

Proof. By definition, and rewriting $|E_o^k|$ as a sum of indicator functions for elements of E:

$$\begin{aligned}\rho_e^{(k)}(G,n_p) &= \mathbb{E}_{\sim V_p}\left[\frac{|E_o^k|}{|E|}\middle||V_p| = n_p\right] \\ &= \frac{1}{|E|}\mathbb{E}_{\sim V_p}\left[\sum_{e\in E} I\{e\in E_o^k\}\middle||V_p| = n_p\right] \\ &= \frac{1}{|E|}\sum_{e\in E}\mathbb{P}_{\sim V_p}\left[e\in E_o^k\middle||V_p| = n_p\right] \\ &= \frac{1}{|E|}\sum_{e\in E}\rho_{e,*}^{(k)}(G,n_p,e)\end{aligned}$$

Theorem 5. Let $G = (V,E)$ be a graph with $n = |V|$ nodes, and $n_p \le n$ a number of primary nodes. Let $e = (u,v)\in E$ be an edge. The $k-$ hop probability of observation of e is given by:

$$\rho_{e,*}^{(k)}(G,n_p,e) = \begin{cases} 1 - \dfrac{C_{n_p}^{n-edgedeg_k(e)}}{C_{n_p}^{n}} & \text{if } edgedeg_k(e)\le n - n_p \\ 1 & \text{otherwise}\end{cases}$$

Where $edgedeg_k(e)$, for $e = (u,v)$, is the $k-$ hop *edge-degree* of *e:*

$$edgedeg_k(e) = |\{w\in V:\ d(u,w)\le k-1 \vee d(v,w)\le k-1\}|$$

Proof. We develop the probabilities, using the definition of E_o and the definition of conditional probabilities:

$$\begin{aligned}\rho_{e,*}^{(k)}(G,n_p,e) &= \mathbb{P}_{\sim V_p}[e\in E_o||V_p| = n_p] \\ &= \mathbb{P}_{\sim V_p\subset V}[\exists w\in V_p : d(u,w)\le k-1\vee d(v,w)\le k-1|V_p| = n_p] \\ &= \mathbb{P}_{\sim V_p\subset V}[\exists w\in V_p : w\in eneigh_k(e)||V_p| = n_p]\end{aligned}$$

Where $eneigh_k(e) = \{w\in V : d(u,w)\le k-1\vee d(v,w)\le k-1\}$. Note that $|eneigh_k(e)| = edgedeg_k(e)$, by definition. We then apply lemma 1 for G and $S = eneigh_k(e)$, which concludes the proof.

Corollary 1. Let $G = (V,E)$ be a graph with $n = |V|$ nodes, and $n_p \le n$ a number of primary nodes. Let $e = (u,v)\in E$ be an edge. The $1-$ hop probability of observation of e is identical for all edges, and given by:

$$\rho_{e,*}^{(1)}(G,n_p,e) = 1 - \frac{n-n_p}{n}\cdot\frac{n-n_p-1}{n-1}$$

Proof. Observe that for $k = 1$ and for any $e = (u,v)\in E$:

$$edgedeg_1(e) = |\{w\in d(u,w)\le 0\vee d(v,w)\le 0\}| = |\{u,v\}| = 2$$

The result then follows from theorem 4.

Datasets

Phone dataset

Our phone dataset comprises 4 weeks of domestic intra-company communications phone logs (calls and texts) of 1.4M customers of a mobile phone provider.

Co-location dataset

The dataset contains mobility information of about 600 students at a European university collected over a month (retrieved via GPS, Wi-Fi, or a combination of the two) along with Bluetooth sensing data (every 5 min), as part of the Copenhagen Networks Study.[31]

Empirical study of observability

We study graphs generated from four models: a complete graph and three random graphs with an average density of 0.015; a Erdős-Rényi graph (with $p = 0.015$), a Barabási-Albert graph (with $m = 2$), and a Watts-Strogatz graph (with $k = 5$ and $p = 0.2$), each with 250 nodes.[17–19] We estimate the node- and edge-observability for each graph type, graph size, and number of primary nodes n_p by selecting 500 random sets V_C of n_p nodes, measuring $|E_o^k|$ and $|V_o^k|$. The curves we report are the average over these sets. To model the influence of graph density on observability (Figure 3), we repeat the same procedure for densities ranging from 0.01 to 0.3, by changing the parameters p, m and k of the graph models.

Observability of cities

In order to estimate the average probability of observation as a function of the number of primary nodes for an area of 1 km^2, we compute hourly graphs from the co-location dataset (Figure 8A), and fit an approximation of the average probability of observation $\frac{1}{|V|}\sum_{u\in V}\rho_{n,*}^{(k)}(G,n_p,u)$ for n_C primary nodes of the form $\hat{\mu}(n_p) = 0.13\log(n_p) - 0.05$ ($R^2 = 0.876$). We use census data to compute population density in blocks of 1 km^2.

Finally, we use algorithm 1 to estimate node-observability of a city when a fraction x of the population is part of the primary data collection, with n_B the number of blocks in a city, and B a list of n_B elements, the population size in each block (given by census data). Algorithm 1 estimates the average probability of observation in each block using the approximation $\hat{\mu}(\circ)$, then scales it to obtain the node-observability (Thm. 1).

Algorithm 1. Node-observability of a city

```
1: procedure NodeObsCity(n_B, B, x)
2:   population ← ∑_i B_i
3:   observed ← 0
4:   for i = 1, …, n_B do
5:     m_i ← x · B_i
6:     observed + = (x + (1 − x) · μ̂(m_i)) · B_i
7:   end for
8:   return observed/population
9: end procedure
```

Sometimes, detailed census data might not be available. Figure 8C also shows that the node-observability in the cities we considered can be reasonably well approximated by using an exponential distribution for the distribution of the population per km^2 in the city (as an approximation of the actual distribution). The parameter λ of the exponential is the density of the entire city $\lambda = \frac{\text{population}}{\text{area}}$. This allows for the global node-observability of a city—the fraction of the population observed by an attacker—to be readily estimated knowing only the population and area of the city.

This extrapolation from the CNS data to cities relies on two assumptions. First, it assumes that the probability for an individual to be observed depends only on the absolute number of primary people it might encounter (n_p) in the cell, and not its total population. Second, it assumes that the mobility of people in the CNS dataset resembles that of the population of cities. This does not account for differences in lifestyle, geography, or population type. However, we believe this to be the first attempt at estimating the risk of mass surveillance in large cities based on actual co-location data, and look forward to future work exploring this question.

SUPPLEMENTAL INFORMATION

Supplemental information can be found online at https://doi.org/10.1016/j.patter.2022.100662.

ACKNOWLEDGMENTS

We would like to thank Sune Lehmann for his invaluable insights. We would like to acknowledge Alan Mislove, Christo Wilson, Arnaud Tournier, Shubham Jain, Andrea Gadotti, and Ali Farzanehfar for their feedback.

AUTHOR CONTRIBUTIONS

Conceptualization, all authors; methodology, all authors; software, L.R. and P.S.; formal analysis, F.H.; investigation, L.R., P.S., F.H., and Y.-A.d.M.; resources, P.S. and Y.-A.d.M.; data curation, P.S. and L.R.; writing – original

CellPress
OPEN ACCESS

draft, L.R., P.S., F.H., and Y.-A.d.M.; writing – review & editing, F.H. and Y.-A.d.M.; visualization, P.S. and F.H.; supervision, Y.-A.d.M. and E.S.; project administration, Y.-A.d.M. and F.H.

DECLARATION OF INTERESTS

The authors declare no competing interests.

Received: July 1, 2022
Revised: August 16, 2022
Accepted: November 29, 2022
Published: January 13, 2023

REFERENCES

1. Castells, M. (2011). The Rise of the Network Society, *12* (John Wiley & Sons).
2. United Nations (2018). 2018 Revision of World Urbanization Prospects.
3. Travers, J., and Milgram, S. (1969). An experimental study of the small world problem. Sociometry *32*, 425–443.
4. Ugander, J., Karrer, B., Backstrom, L., and Marlow, C. (2011). The anatomy of the facebook social graph. Preprint at arXiv. https://doi.org/10.48550/arXiv.1111.4503.
5. Backstrom, L., Boldi, P., Rosa, M., Ugander, J., and Vigna, S. (2012). Four degrees of separation. In Proceedings of the 4th Annual ACM Web Science Conference, pp. 33–42.
6. Edunov, S., Diuk, C., Filiz, I.O., Bhagat, S., and Burke, M. (2016). Three and a Half Degrees of Separation (Research at Facebook).
7. Bettencourt, L.M.A. (2013). The origins of scaling in cities. Science *340*, 1438–1441.
8. Bettencourt, L.M.A., Lobo, J., Helbing, D., Kühnert, C., and West, G.B. (2007). Growth, innovation, scaling, and the pace of life in cities. Proc. Natl. Acad. Sci. USA *104*, 7301–7306.
9. Carlino, G.A., Chatterjee, S., and Hunt, R.M. (2007). Urban density and the rate of invention. J. Urban Econ. *61*, 389–419.
10. Pickard, G., Pan, W., Rahwan, I., Cebrian, M., Crane, R., Madan, A., and Pentland, A. (2011). Time-critical social mobilization. Science *334*, 509–512.
11. Khatib, F., DiMaio, F., Foldit Contenders Group; Foldit Void Crushers Group, Cooper, S., Kazmierczyk, M., Gilski, M., Krzywda, S., Zabranska, H., Pichova, I., et al. (2011). Crystal structure of a monomeric retroviral protease solved by protein folding game players. Nat. Struct. Mol. Biol. *18*, 1175–1177.
12. Lazer, D., Pentland, A., Adamic, L., Aral, S., Barabási, A.L., Brewer, D., Christakis, N., Contractor, N., Fowler, J., Gutmann, M., et al. (2009). Life in the network: the coming age of computational social. Science *323*, 721–723.
13. Eddington, P.G. (2019). The snowden effet, six years on. Just Security. https://www.justsecurity.org/64464/the-snowden-effect-six-years-on/.
14. Lapowsky, I. (2019). How cambridge analytica sparked the great privacy awakening. Wired. https://www.wired.com/story/cambridge-analytica-facebook-privacy-awakening/.
15. European Data Protection Supervisor (2019). Edps guidelines on assessing the proportionality of measures that limit the fundamental rights to privacy and the protection of personal data. https://edps.europa.eu/sites/edp/files/publication/19-12-19_edps_proportionality_guidelines2_en.pdf.
16. Venkatadri, G., Lucherini, E., Sapiezynski, P., and Mislove, A. (2018). Investigating sources of pii used in facebook's targeted advertising. Proceedings on Privacy Enhancing Technologies 2019, 227–244.
17. Barabási, A.-L., and Albert, R. (1999). Emergence of scaling in random networks. Science *286*, 509–512.
18. Watts, D.J., and Strogatz, S.H. (1998). Collective dynamics of 'small-world' networks. Nature *393*, 440–442.
19. Erdos, P., and Rényi, A. (1960). On the evolution of random graphs. Publ. Math. Inst. Hung. Acad. Sci *5*, 17–60.
20. Cadwalladr, C., and Graham-Harrison, E. (2018). Revealed: 50 million facebook profiles harvested for cambridge analytica in major data breach. The Guardian. https://www.theguardian.com/news/2018/mar/17/cambridge-analytica-facebook-influence-us-election.
21. Schroepfer, M. (2018). An update on our plans to restrict data access on facebook. Meta. https://newsroom.fb.com/news/2018/04/restricting-data-access/.
22. Davies, R., and Rushe, D. (2019). Facebook to pay $5bn fine as regulator settles cambridge analytica complaint. The Guardian. https://www.theguardian.com/technology/2019/jul/24/facebook-to-pay-5bn-fine-as-regulator-files-cambridge-analytica-complaint.
23. Barabási, A.-L. (2016). Network Science (Cambridge university press). http://networksciencebook.com/.
24. Rose, J., Barton, C., Souza, R., and Platt, J. (2014). Data Privacy by the Numbers. Boston Consulting Group. https://www.bcg.com/publications/2014/data-privacy-numbers.
25. McCarthy, M.T. (2002). USA PATRIOT Act.
26. Radio France (2018). Au coeur de l'antenj, la cellule des écoutes téléphoniques judiciaires. https://www.franceinter.fr/emissions/dans-le-pretoire/dans-le-pretoire-27-avril-2018.
27. Kelly, J. (2017). Enforcement of the Immigration Laws to Serve the National Interest (Department of Homeland Security).
28. Mayer, J., Mutchler, P., and Mitchell, J.C. (2016). Evaluating the privacy properties of telephone metadata. Proc. Natl. Acad. Sci. USA *113*, 5536–5541.
29. Aiello, W., Chung, F., and Lu, L. (2000). A random graph model for massive graphs. In STOC, *2000*, pp. 1–10.
30. Domanski, H. (2017). This flashlight app for android wants to steal all your money. TechRadar. http://www.techradar.com/news/this-flashlight-app-for-android-wants-to-steal-all-your-money.
31. Stopczynski, A., Sekara, V., Sapiezynski, P., Cuttone, A., Madsen, M.M., Larsen, J.E., and Lehmann, S. (2014). Measuring large-scale social networks with high resolution. PLoS One *9*, e95978.
32. Humbert, M., Trubert, B., and Huguenin, K. (2019). A survey on interdependent privacy. ACM Comput. Surv. *52*, 1–40.
33. Floridi, L. (2014). Open data, data protection, and group privacy. Philos. Technol. *27*, 1–3.
34. Mislove, A., Viswanath, B., Gummadi, K.P., and Druschel, P. (2010). You are who you know: inferring user profiles in online social networks. In Proceedings of the Third ACM International Conference on Web Search and Data Mining, WSDM '10, pp. 251–260. https://doi.org/10.1145/1718487.1718519.
35. Lampinen, A. (2015). Networked privacy beyond the individual: four perspectives to'sharing'. In Proceedings of The Fifth Decennial Aarhus Conference on Critical Alternatives (Aarhus University Press), pp. 25–28.
36. Li, N., Li, T., and Venkatasubramanian, S. (2007). t-closeness: privacy beyond k-anonymity and l-diversity. In Data Engineering, 2007. ICDE 2007. IEEE 23rd International Conference on, IEEE, pp. 106–115.
37. Narayanan, A., and Shmatikov, V. (2005). Obfuscated databases and group privacy. In Proceedings of the 12th ACM conference on Computer and communications security, pp. 102–111.
38. Callaway, D.S., Newman, M.E., Strogatz, S.H., and Watts, D.J. (2000). Network robustness and fragility: percolation on random graphs. Phys. Rev. Lett. *85*, 5468–5471.
39. Shang, Y., Luo, W., and Xu, S. (2011). L-hop percolation on networks with arbitrary degree distributions and its applications. Phys. Rev. E *84*, 031113.
40. McGoogan, C. (2017). Millions of android devices infected with malware in popular judy game. The Telegraph. http://www.telegraph.co.uk/technology/2017/05/31/millions-android-devices-infected-malware-popular-judy-game/.

41. Federal Trade Commission (2014). FTC approves final order settling charges against flashlight app creator. https://www.ftc.gov/news-events/press-releases/2014/04/ftc-approves-final-order-settling-charges-against-flashlight-app.

42. Manthorpe, R. (2018). Sam amrani tracks you in pret. and at starbucks. and down the pub. Wired. https://www.wired.co.uk/article/tamoco-sam-amrami-proximity-tracking-mwc.

43. Tau, B. (2020). Next step in government data tracking is the internet of things, the Wall Street Journal. The Wall Street Journal. https://www.wsj.com/articles/next-step-in-government-data-tracking-is-the-internet-of-things-11606478401.

44. Bergman, R., and Mazzetti, M. (2022). The battle for the world's most powerful cyberweapon. The New York Times. https://www.nytimes.com/2022/01/28/magazine/nso-group-israel-spyware.html.

45. European Commission (2020). Coronavirus: an EU approach for efficient contact tracing apps to support gradual lifting of confinement measures. https://ec.europa.eu/commission/presscorner/detail/en/ip_20_670.

46. Jain, S., Bensaid, E., and de Montjoye, Y.-A. (2019). UNVEIL: capture and visualise WiFi data leakages. In The World Wide Web Conference (ACM), pp. 3550–3554.

47. Sapiezynski, P., Gatej, R., Mislove, A., and Lehmann, S. (2015). Opportunities and challenges in crowdsourced wardriving. In Proceedings of the 2015 ACM Conference on Internet Measurement Conference (ACM), pp. 267–273.

48. Sapiezynski, P., Stopczynski, A., Gatej, R., and Lehmann, S. (2015). Tracking human mobility using wifi signals. PLoS One *10*, e0130824.

49. Android (2022). MAC randomization behavior: non-persistent randomization. https://source.android.com/docs/core/connect/wifi-mac-randomization-behavior#non-persistent.

50. Troncoso, C., Payer, M., Hubaux, J.-P., Salathé, M., Larus, J., Bugnion, E., Lueks, W., Stadler, T., Pyrgelis, A., Antonioli, D., et al. (2020). Decentralized privacy-preserving proximity tracing. Preprint at arXiv. https://doi.org/10.48550/arXiv.2005.12273.

51. Dehaye, P.-O., and Reardon, J. (2020). Proximity tracing in an ecosystem of surveillance capitalism. In Proceedings of the 19th Workshop on Privacy in the Electronic Society, pp. 191–203.

52. Sapieżyński, P., Stopczynski, A., Lassen, D.D., and Lehmann, S. (2019). Interaction data from the Copenhagen Networks Study. Sci. Data *6*. 315.

Patterns, Volume *4*

Supplemental information

Detrimental network effects in privacy: A graph-theoretic model for node-based intrusions

Florimond Houssiau, Piotr Sapieżyński, Laura Radaelli, Erez Shmueli, and Yves-Alexandre de Montjoye

Supplemental Experimental Procedures

***S1**: Alternatives to uniform node intrusions*

In this paper, we study edge- and node-observability through uniform node-based intrusions. While this assumption is realistic for the three use cases we present, other non-uniform attacks would be interesting to investigate. Most of our mathematical definitions do not rely on this uniformity assumption and the framework can be easily extended with different compromised nodes distribution or targeting. For instance, targeted attacks to observe the entire network using as few nodes as possible have been studied before [1, 2]. These differ from our work in that the attacker knows the structure of entire network, and chooses nodes to compromise in order to learn all of the nodes' private states (e.g. in a power grid).

***S2**: Facebook's node-observability*

Our analysis of Facebook's node-observability is based on the degree distribution of Facebook users in the United States in 2011 [3]. We shift and truncate this distribution so that the average degree matches the 2014's average degree (which has been published [4]). Formally, let $P_{2011}(d)$ be the distribution of degrees in 2011, such that $\sum_{d=0}^{5000} P_{2011}(d) = 1$. This distribution is truncated at 5000 because Facebook doesn't allow a user to have more than that number of friends. We compute an estimate degree distribution for 2014 as:

$$\widehat{P}_{2014}(d) = \begin{cases} 0 & \text{if } d < \sigma \\ A \cdot P_{2011}(d - \sigma) & \text{otherwise} \end{cases}$$

here the shift σ is chosen such that the average degree $\sum_{d=0}^{5000} d\widehat{P}_{2014}(d)$ matches the empirical value, and the scaling constant A is such that $\widehat{P}_{2014}$ sums to 1. This is a simple approximation, assuming that users uniformly increased their friends count between 2011 and 2014.

We convert the distribution $\widehat{P}_{2014}$ to absolute degree counts by multiplying by $N = 205 \cdot 10^6$, the number of Facebook users in the USA [5]. We then use theorem 3 to compute the 1-hop node-observability from the degree counts. Our analysis shows that compromising $n_C = 270,000$ profiles, as Cambridge Analytica did, allows an attacker to observe 68.0 million users, giving a node-observability of 0.318.

To study the 2-hops observability of the Facebook graph, we generate synthetic graphs from the distribution $\hat{P}_{2014}$ using the configuration model [6]. Generating such a graph with $N = 205 \cdot 10^6$ nodes is impractical, we compute the observability curve for graphs with $N' < N$ nodes, with N' ranging from 10^5 to $2 \cdot 10^6$, shown in Fig. 1. Our results show that compromising a fraction $\frac{n_{CA}}{\text{US population}} = \frac{270000}{205 \cdot 10^6} \approx 0.0013$ of the network would leads to a node-observability of 1.0 on all the synthetic graphs. This analysis strongly suggests that a 2-hop policy would have enabled Cambridge Analytica to observe the whole Facebook network.

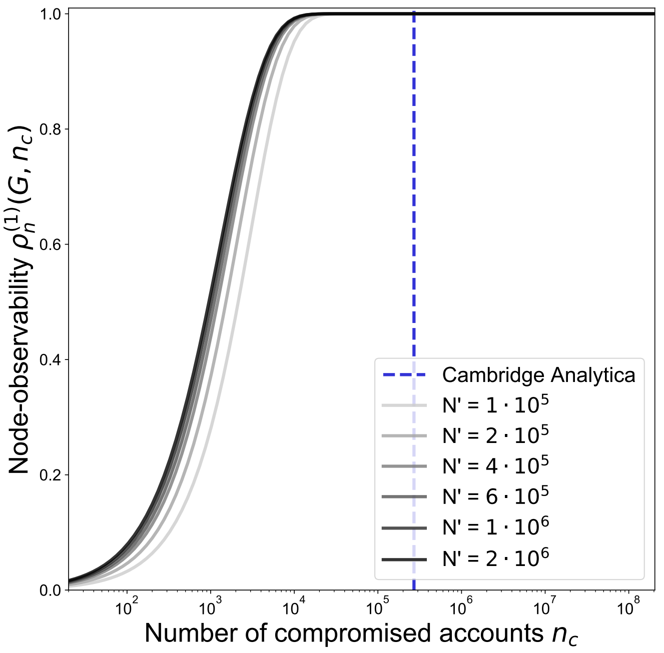

Figure 1: **Estimation of Cambridge Analytica's attack success if Facebook allowed for 2-hop**. 2-hop node-observability of graphs generated using the configuration model with degree distribution similar to that of the shifted Facebook degree distribution $\hat{P}_{2014}$ (solid curve). The curves correspond to graphs with different number of nodes N' smaller than $N = 205 \cdot 10^6$, the number of Facebook users in the USA in 2014. The curves quickly converge for growing n', and all agree for $n_p = 270,000$ (Cambridge Analytica).

***S3**: Close proximity Networks*

The first step of our computation of the observability of cities is to estimate the average probability of observation from a fixed number of sensors in a 1 km^2 cell. We use Algorithm 1 to estimate this from geo-tagged hourly co-location data, which we model as a function of the hourly time-stamp (in a set of hours $\mathcal{T}$) and the cell (from a set of 1 km^2 cells $\mathcal{C}$) to the set of all graphs (denoted by $\mathbb{G}$) $\mathbb{G} : \mathcal{T} \times \mathcal{C} \to \mathbb{G}$.

Algorithm 1 Average Probability of Observation from hourly co-location data

procedure AVGPROBAOBS($n_p, \mathbb{G}, n_{samples}$)
  $observed \leftarrow 0$
  for $i = 1, \ldots, n_{samples}$ **do**
    Pick a cell uniformly at random $c \leftarrow \mathcal{C}$;
    Pick a time uniformly at random $h \leftarrow \mathcal{T}$;
    Define $G(c, h) = (V, E)$, pick n_p nodes uniformly at random from V, V_p;
    Pick a target u uniformly at random from non-infected nodes $u \leftarrow V \backslash V_p$;
    if there is an edge between u and a node in V_p **then**
      $observed += 1$
    end if
    $m_i \leftarrow x \cdot B_i$
  end for
  return $observed/n_{samples}$
end procedure

We use procedure AvgProbaObs with n_p ranging from 5 to 385, in increments of 5, with $n_{samples} = 25$ (about 10000 total samples). Fig. 2 presents our results, along with the fit by $\hat{\mu}(n_p) = 0.13 \log(n_p) - 0.05$ ($R^2 = 0.876$).

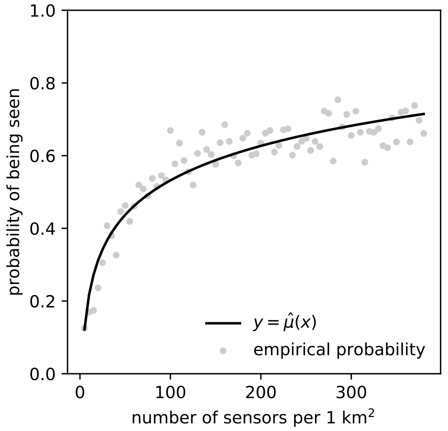

Figure 2: **Average probability of observation in hourly co-location graphs in a** $1\ km^2$ **cell.** Each dot is obtained from AvgProbaObs on the DTU dataset, using $n_{samples} = 25$. The solid curve is $\hat{\mu}(n_p) = 0.13 \log(n_p) - 0.05$, which fits the observed data ($R^2 = 0.876$).

References

[1] Yang Yang, Jianhui Wang, and Adilson E Motter. Network observability transitions. *Physical Review Letters*, 109(25):258701, 2012.

[2] Takehisa Hasegawa, Taro Takaguchi, and Naoki Masuda. Observability transitions in correlated networks. *Physical Review E*, 88(4):042809, 2013.

[3] Johan Ugander, Brian Karrer, Lars Backstrom, and Cameron Marlow. The anatomy of the facebook social graph. *arXiv preprint arXiv:1111.4503*, (2011).

[4] Aaron Smith. What people like and dislike about facebook, February 2014.

[5] Statista. Number of monthly active facebook users in the united states and canada as of 4th quarter 2018 (in millions)., 2018.

[6] Mark EJ Newman. The structure and function of complex networks. *SIAM review*, 45(2):167–256, 2003.
